# AGE, AGING, AND SOCIAL JUSTICE

**DOI:** 10.1093/geroni/igae098.0179

**Published:** 2024-12-31

**Authors:** Jan Baars

**Affiliations:** University for Humanistic Studies, Haarlem, Noord-Holland, Netherlands

## Abstract

The concept of Emancipatory Gerontology goes back to the early work of Habermas. An emancipatory human interest was presented as constitutive for a critical social science that reflects on its own social and methodological presuppositions and on the practical consequences of scientifically based actions. This program has lost nothing of its urgency. Studies of aging need to consider that they are part of a society where Long Lives are for the Rich (Baars, 2024). Even in a relatively egalitarian society as The Netherlands persons with a low SES live eight years shorter than the more advantaged and live 23 years longer with chronic disease. This widely spread phenomenon in Western countries qualifies not only institutionalized aging processes as deeply unequal but also changes the significance of ‘middle age’, education or work. Studies of aging cohorts need to include those who have already died of causes that can hardly be seen as autonomous choice or genetic misfortune. This profound inequality over the life course is constituted by a neoliberal society in which the rich become richer and the life course is increasingly organized as an entrepreneurial challenge. Besides the injustice that is inflicted upon disadvantaged citizens these structural inequalities are beginning to rip societies apart. However, social justice theories of aging, with their naturalization of ‘age’ and ‘age groups’ have hardly noticed the effects of social inequality on aging processes. These shortcomings will be analyzed and confronted with more adequate theories of social justice and aging.

